# PvrA-Mediated Inhibition of Choline and Ethanolamine Uptake Promotes *Pseudomonas aeruginosa* Colonization in the Host Environment

**DOI:** 10.3390/pathogens15070680

**Published:** 2026-06-26

**Authors:** Shuo Wang, Liwen Yin, Jinhao Yang, Changru Zhang, Zhi Yao, Xiaolei Pan

**Affiliations:** 1 State Key Laboratory of Experimental Hematology, Key Laboratory of Immune Microenvironment and Disease (Ministry of Education), Tianjin Institute of Immunology, Department of Immunology, School of Basic Medical Sciences, Tianjin Medical University, Tianjin 300070, China; shuowang2000@163.com (S.W.); yangjinhao0821@tmu.edu.cn (J.Y.); zcr20030818@163.com (C.Z.); 2 State Key Laboratory of Medicinal Chemical Biology, Key Laboratory of Molecular Microbiology and Technology of the Ministry of Education, Department of Microbiology, College of Life Sciences, Nankai University, Tianjin 300071, China; yinliwen2022@163.com

**Keywords:** *P. aeruginosa*, PvrA, fatty acids

## Abstract

*Pseudomonas aeruginosa* can utilize abundant phosphatidylcholine (PC) and phosphatidylethanolamine (PE) within the host as energy and structural substrates. Fatty acids, choline, and ethanolamine liberated from PC and PE can each serve as the sole carbon source to support bacterial growth in vitro. Our previous work demonstrated that fatty acid metabolism is critical for acute pulmonary infection caused by *P. aeruginosa*. The pathogen senses host-derived fatty acids via the transcriptional regulator PvrA, which activates fatty acid utilization pathways and drives the production of virulence factors required for acute infection. In this study, we demonstrate that during acute pulmonary infection in mice, *P. aeruginosa* upregulates fatty acid catabolism while simultaneously repressing choline and ethanolamine uptake and metabolism pathways. Deletion of the transcriptional activators GbdR and EatR (which control choline and ethanolamine utilization respectively) enhances pulmonary bacterial colonization. We further identify fatty acids as environmental signals that trigger repression of choline and ethanolamine utilization programs. PvrA mediates this signaling cascade by directly binding to the promoters of *gbdR* and *eatR* and suppressing their transcription upon fatty acid exposure.

## 1. Introduction

*Pseudomonas aeruginosa* is an opportunistic human pathogen that commonly infects immunocompromised individuals, particularly patients with cystic fibrosis (CF) or chronic obstructive pulmonary disease (COPD) [1,2,3,4]. Its exceptional metabolic plasticity and broad-spectrum intrinsic/acquired multidrug resistance render such infections difficult to eradicate [5,6,7]. This bacterium preferentially colonizes the respiratory tract, where PC and PE enriched in pulmonary surfactant act as key carbon reservoirs during infection [4,8,9,10]. Secreted phospholipases and esterases hydrolyze PC and PE to release long-chain fatty acids, glycerol, phosphocholine and phosphoethanolamine [9,10]. Internalized fatty acids undergo β-oxidation to generate energy and precursors for virulence factor synthesis. The transcriptional regulators PsrA, PvrA and DesT are known to mediate fatty acid-dependent transcriptional responses in *P. aeruginosa* [11,12,13,14,15,16,17]. Glycerol, ethanolamine and choline released from phospholipid breakdown each possess dedicated uptake and degradation pathways governed by distinct transcriptional regulators. Glycerol is processed and incorporated into central carbon metabolism following internalization [18,19]. Ethanolamine assimilation relies on the Eat transporter and EutBC catabolic enzymes, with the corresponding operon transcriptionally activated by EatR [20,21]. Choline is imported via CbcWV or BetT transporters and subsequently converted to glycine betaine for osmotic protection and further catabolism, with GbdR acting as the master transcriptional activator of choline utilization genes [22,23,24,25]. Here, we demonstrate that during acute pulmonary infection in a murine model, *P. aeruginosa* detects host fatty acids through PvrA, which elicits transcriptional repression of *eatR* and *gbdR* and consequently suppresses ethanolamine and choline utilization.

## 2. Materials and Methods

### 2.1. Bacterial Strains, Plasmids and Culture Media

All bacterial strains and plasmids used in this study are listed in Table S1. Bacterial cells were grown in Luria–Bertani (LB) broth (10 g/L tryptone, 5 g/L yeast extract, 5 g/L NaCl, pH 7.4). The low phosphate medium is composed of 20 g of pancreatic digest of gelatin (Difco, Franklin Lakes, NJ, USA), 0.14 g MgCl_2_, and 10 g K_2_SO_4_ per liter of H_2_O. Medium with sole carbon source was prepared in 1× M9 medium (49.4 mM Na_2_HPO_4_, 24.0 mM KH_2_PO_4_, 9 mM NaCl, 19 mM NH_4_Cl, 0.5 mM MgSO_4_) with 0.2% (*w*/*v*) Brij-58, supplemented with 0.1% (*w*/*v*) glucose or 0.4% (*w*/*v*) PC, PE or palmitic acid, resulting in GC-M9, PC-M9, PE-M9 or FA-M9 [9,16,26,27].

### 2.2. RNA Isolation and Quantitative Real Time PCR

Bacterial cultures were harvested at indicated growth phases in LB medium or indicated mediums. Total bacterial RNA was extracted using the RNAprep pure cell/bacteria kit (Tiangen Biotec, Beijing, China) following the manufacturer’s instructions. The cDNA was synthesized from 1 μg RNA with random primers and a reverse transcriptase (TAKARA, Dalian, China). Quantitative real time PCR (qRT-PCR) was performed by using a CFX Connect Real-Time system (Bio-Rad, Hercules, CA, USA). The indicated specific primers are listed in Table S1. Amplification efficiency for each primer pair was evaluated using 2-fold serial dilutions of pooled cDNA. Standard curves were generated by plotting Ct values against the logarithm of cDNA concentration. Primer efficiency was calculated as E = (10^−1/slope^ − 1) × 100%. Primers with amplification efficiency between 90% and 110% and R^2^ > 0.99 were used for subsequent experiments. The indicated specific primers, cDNA and a SYBR II green supermix (Bio-Rad, Beijing, China) were mixed in a total volume of 20 μL. The relative gene expression level was calculated using the 2^−ΔΔCt^ method with the 30S ribosomal protein gene *rpsL* as the internal reference. The expression stability of the reference gene *rpsL* was validated across all experimental groups. The Ct values of *rpsL* from all samples were collected and analyzed via ΔCt method. The low variation in Ct values and stability index confirmed that *rpsL* was stably expressed under all tested conditions and suitable as the reference gene for qRT-PCR normalization. Data represent the mean ± standard deviation of the results from three biological replicates.

### 2.3. Animal Experiment

All the animal assays were carried out in accordance with National guidelines on the use of animals in research. The protocols were approved by the Institutional Animal Care and Use Committee of Tianjin Medical University (approval number: TMUaMEC 2024057).

The murine acute pneumonia model was performed as previously described with minor modifications [28]. In brief, overnight bacterial cultures were grown to an OD_600_ of 1.0 at 37 °C in LB. The bacteria were collected by centrifugation at 12,000 *g* for 1 min, followed by washing with PBS twice. The bacteria were then resuspended in PBS to a concentration of 1 × 10^8^ colony-forming units (CFU)/mL. Female BALB/c mice aged 6–8 weeks with matched body weight were randomly assigned to each experimental group using a random number generator, with eight mice per group to ensure sufficient statistical power for detecting biologically meaningful intergroup differences. After being anesthetized through intraperitoneal injection of 80–100 μL of 7.5% chloral hydrate, each mouse was intranasally inoculated with 20 μL of the bacterial suspension. At 12 h post-infection (hpi) and 24 hpi, the mice were sacrificed by CO_2_. Bacteria in the bronchi alveolar lavage fluid (BALF) were collected by centrifugation at 12,000 *g* for 2 min. The total RNA was isolated using Trizol Reagent (Thermo Fisher Scientific, Waltham, MA, USA). As for bacterial colonization determination, the lungs were harvested and homogenized in 1% peptone and bacterial loads were determined by plating. All procedures including tissue dissection, sample processing, and colony counting were performed by investigators blinded to group allocations to eliminate subjective assessment bias and improve experimental transparency.

### 2.4. β-Galactosidase Assay

The β-galactosidase assay was performed as previously described with modifications [29]. Overnight bacterial cultures were diluted 1:100 to fresh LB. After grown to an OD_600_ of 1.0, 0.5 mL of the bacteria culture was collected by centrifugation and resuspended in 1.5 mL Z buffer (0.06 M Na_2_HPO_4_, 0.04 M NaH_2_PO_4_, 0.01 M KCl, 0.001 M MgSO_4_ and 0.05 M β-mercaptoethanol). A total of 1 mL of the bacterial suspension was used to measure the cell density at OD_600_. To 0.5 mL of the bacterial suspension, 10 μL 0.1% sodium dodecyl sulfate (SDS) and 10 μL chloroform were added, followed by vortex for 10 s to break up the cells. A total of 0.1 mL orthonitrophenyl-galactopyranoside (ONPG, 4 mg/mL) (BBI life sciences, Shanghai, China) was added to initiate the reaction at 37 °C, 500 μL 1 M Na_2_CO_3_ was added to stop the reaction and the reaction time was recorded. After centrifugation, the suspension was used to measure the color development of the samples at OD_420_. The β-galactosidase activities (Miller units) were calculated as follows: Miller units = (1000 × OD_420_)/T/0.5/OD_600_; T, reaction time.

### 2.5. Electrophoretic Mobility Shift Assay

EMSA assays were performed as described previously with minor modifications [16]. DNA probes were amplified by PCR using primers in Table S1. Recombinant PvrA protein was purified via nickel affinity chromatography. Indicated DNA fragments (50 ng) were incubated with purified protein at various concentrations in a 20 µL reaction buffer (4% glycerol, 10 mM pH 7.6 Tris-HCl, 5 mM CaCl_2_, 100 mM NaCl, 1 mM EDTA, and 10 mM-β-mercaptoethanol). Reaction mixtures were kept on ice for 20 min, followed by a 5 min incubation at 37 °C. Samples were then loaded on an 8% native polyacrylamide Tris-borate-EDTA (TBE) gel that had been pre-run at 120 V for 1 h. Electrophoresis was performed at a constant current of 10 mA for 1 h on ice. DNA bands were stained with ethidium bromide (0.5 µg/mL) and visualized with the molecular imager ChemiDoc XRS+ (Bio-Rad, Hercules, CA, USA).

### 2.6. Chromatin Immunoprecipitation

ChIP was performed as previously described with minor modifications [16]. Briefly, *P. aeruginosa* harboring the Flag-tagged PvrA were harvested and treated with formaldehyde to cross-link proteins with DNA. The DNA was sonicated to sizes of 200–500 bp. A total of 2% of the supernatant was saved as input and 100 mL of the supernatant was incubated with 10 g anti-Flag antibody (catalog No. MAB3118, MilliporeSigma, St. Louis, MO, USA). ChIP-qPCR was run on a CFX Connect Real-Time system (Bio-Rad, Hercules, CA, USA) using dedicated primers, and fold enrichment was normalized to input control.

### 2.7. Statistical Analysis

All statistical analyses were performed using dedicated statistical software (GraphPad Prism 10.6.1). Data normality was assessed prior to intergroup comparisons. For datasets conforming to normal distribution, parametric statistical tests were applied; non-parametric tests were used for non-normally distributed data accordingly. The selection of specific statistical methods was clearly stated alongside each corresponding result. Sample sizes for cell culture assays and animal experiments were predefined based on preliminary experimental variation and expected biological effect magnitudes, ensuring sufficient statistical power to identify biologically relevant differences between experimental groups. All statistical tests were two-sided, and statistical significance was defined at *p* < 0.05 unless otherwise specified.

## 3. Results

### 3.1. Ethanolamine and Choline Utilization Genes Are Repressed During Acute Pulmonary Infection

**Figure 1:**
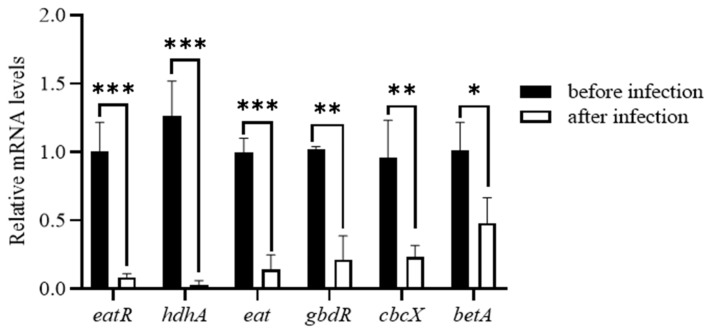
Relative mRNA levels before and after infection were determined by RT-qPCR. The fold changes were calculated relative to the expression level before infection. Data represent the mean ± standard deviation of the results from three samples. *, *p* < 0.05; **, *p* < 0.01; ***, *p* < 0.001 by Student’s *t*-test.

To investigate the infection process of *P. aeruginosa*, we previously generated a transcriptional profile of the bacterium in a murine model of acute pneumonia [16]. Genes involved in ethanolamine uptake and metabolism (PA4021–PA4025) and choline metabolism (*gbdR*, *betAB*, c*bcX*) were downregulated (Table 1), a phenotype independently validated by RT-qPCR (Figure 1). Given that ethanolamine and choline can act as carbon substrates for *P. aeruginosa*, the reduced transcription of their metabolic genes reflects a transcriptional regulatory mechanism that suppresses these two utilization pathways during infection.

### 3.2. Mutants Deficient in Ethanolamine and Choline Utilization Display Enhanced Lung Colonization Capacity

**Figure 2:**
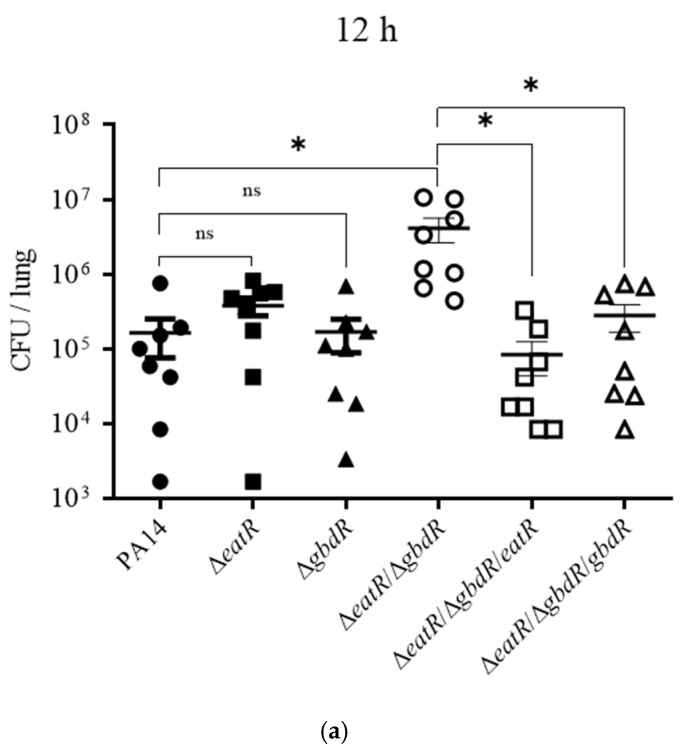

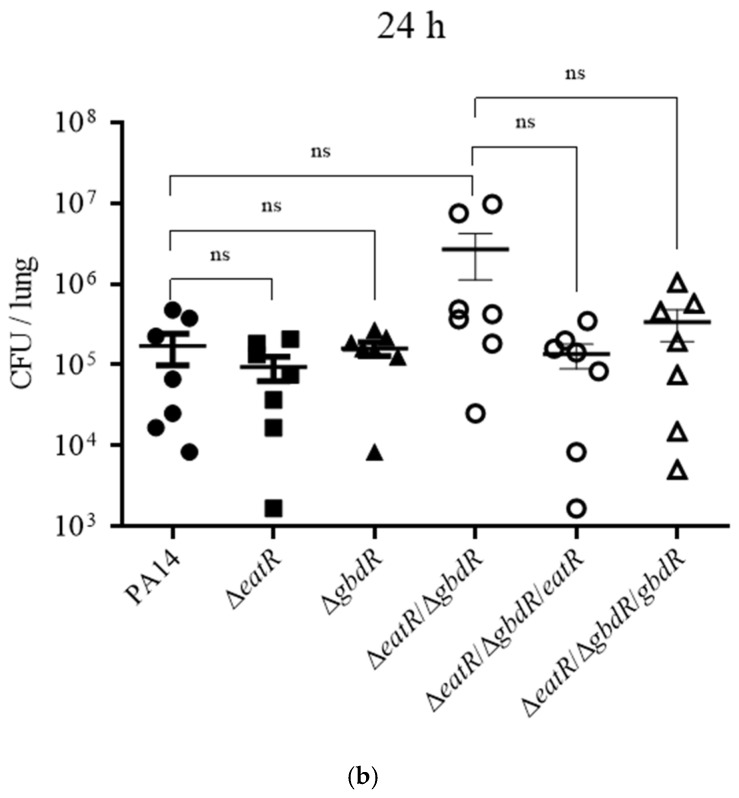
Mice received intranasal instillation of 2 × 10^6^ CFU of the indicated strains. At 12 h (**a**) and 24 h (**b**) post-infection, the mice were sacrificed and lungs were isolated and homogenized. The bacterial loads were determined by serial dilution and plating. *, *p* < 0.05; ns, not significant by the Mann–Whitney test.

We hypothesized that repression of ethanolamine and choline metabolism may benefit bacterial adaptation. To test this, we constructed single Δ*eatR*, Δ*gbdR* knockouts and a Δ*eatR*Δ*gbdR* double mutant and quantified pulmonary bacterial burdens at 12 and 24 hpi in the murine pneumonia model. Individual deletion of either transcriptional regulator exerted no significant impact on lung colonization, whereas simultaneous deletion of both genes markedly elevated colonization. Genetic complementation of either gene in the double mutant restored bacterial loads to levels comparable to wild-type or single-mutant strains (Figure 2). This phenotypic shift correlates with the loss of ethanolamine and choline metabolism pathways. Multiple indirect factors, including altered central metabolism and cellular stress responses, may also contribute to the observed colonization differences.

### 3.3. Fatty Acids Act as the Upstream Signal Driving Choline and Ethanolamine Catabolic Repression

Our prior transcriptomic analyses demonstrated that fatty acid catabolic machinery is transcriptionally upregulated during murine pulmonary infection, with PvrA functioning as the master sensor of host fatty acid cues (Table 2) [16].

**Figure 3:**
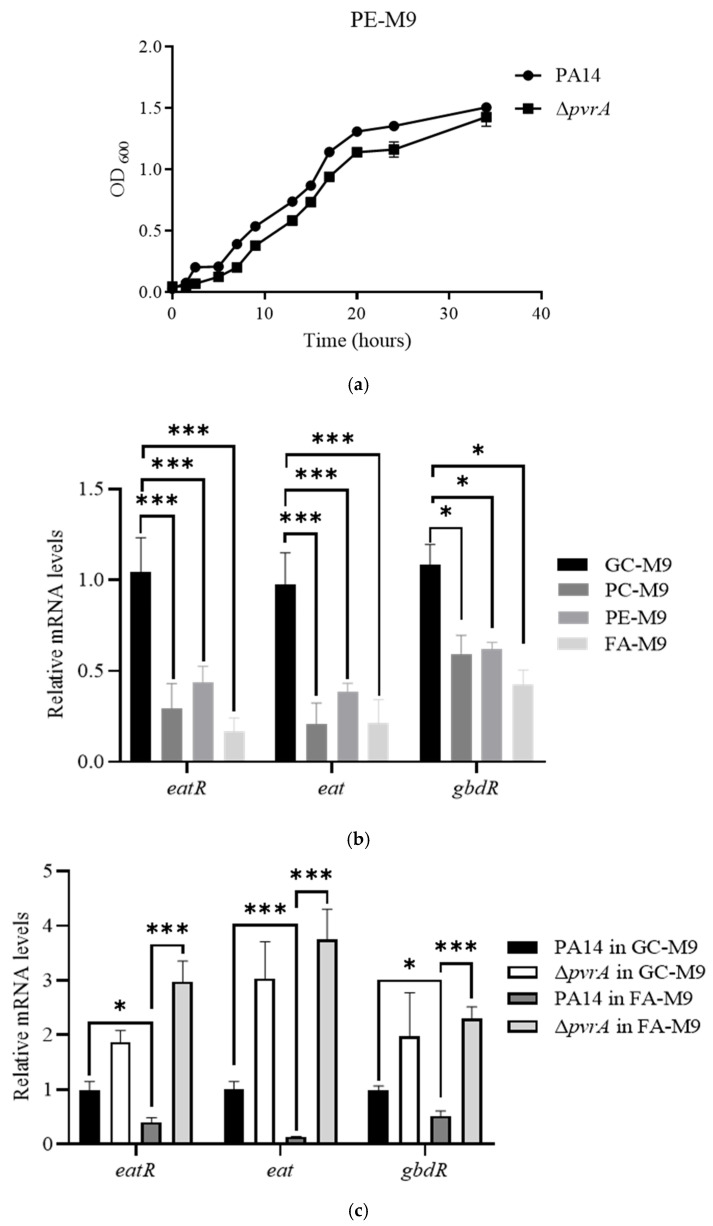
(**a**) The bacterial growth of PA14 and Δ*pvrA* grown in PE-M9 was monitored by measuring OD_600_. (**b**) PA14 was inoculated into minimal media supplemented with glucose (GC), PC, PE or fatty acid (FA) as the sole carbon source and grown to an OD_600_ of 1.0. The mRNA levels of *eatR*, *eat*, and *gbdR* were determined by RT-qPCR. (**c**) The mRNA levels of *eatR*, *eat*, and *gbdR* in PA14 and Δ*pvrA* grown in GC and FA to OD_600_ as 1.0 were determined by RT-qPCR. Data represent the means from three independent experiments, and error bars indicate standard deviations. *, *p* < 0.05; ***, *p* < 0.001 by Student’s *t*-test.

Within the pulmonary surfactant microenvironment, PC and PE are co-hydrolyzed to release fatty acids alongside choline and ethanolamine, prompting our hypothesis that fatty acids constitute the primary repressive metabolic signal. To verify this, we cultured wild-type PA14 in minimal media supplemented with glucose (GC-M9), PC (PC-M9), PE (PE-M9), or fatty acids (FA-M9) as the sole carbon source. Growth curves of PA14 cultured in GC-M9, PC-M9 and FA-M9 have been previously documented [16]; here we report growth curves in PE-M9 minimal media were performed (Figure 3a). All cultures were harvested at OD_600_ = 1.0 (mid-exponential phase) for qRT-PCR analysis to standardize the growth phase across all groups and minimize growth-rate-mediated interference on gene expression profiling. Compared to glucose-grown cells, PA14 cultured in PC, PE, or fatty acids showed downregulation of ethanolamine and choline metabolism genes (Figure 3b). Since both PC and PE liberate free fatty acids upon bacterial hydrolysis, these data suggest that fatty acids may trigger the transcriptional repression of ethanolamine and choline metabolism pathways. Notably, in the Δ*pvrA* mutant, expression of these genes in fatty acid-containing medium was comparable to glucose-cultured controls and significantly higher than wild-type levels (Figure 3c), confirming PvrA is required for fatty acid-mediated transcriptional suppression.

### 3.4. PvrA Directly Represses Transcriptional Activators of Ethanolamine and Choline Utilization

**Figure 4:**
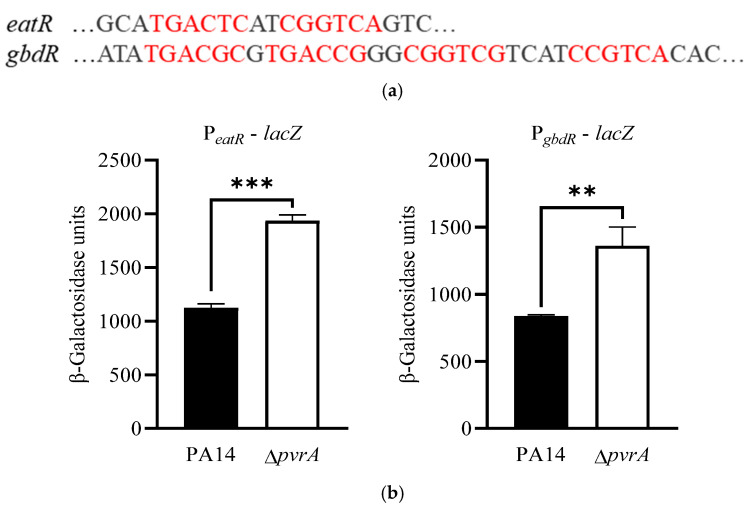

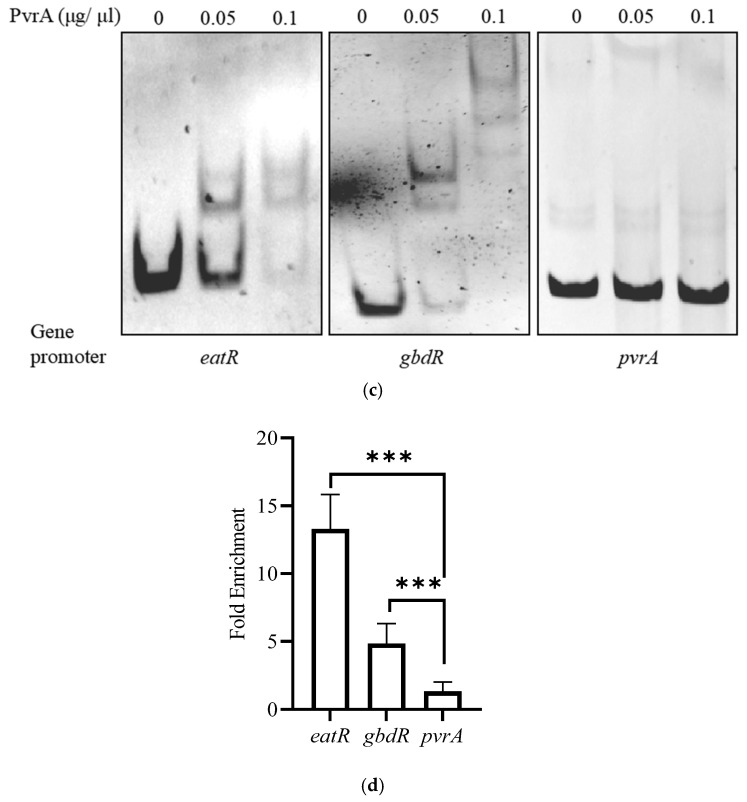
(**a**) Conserved PvrA binding sites in the promoter regions of *eatR* and *gbdR* are shown in red. (**b**) Promoter activities of *eatR* and *gbdR* were determined by β-galactosidase assay. (**c**) The purified PvrA was incubated with indicated DNA probes. The samples were subjected to electrophoresis in a native gel, followed by staining. The promoter of *pvrA* was used as a negative control. (**d**) The presence of the *eatR* and *gbdR* promoters harboring potential PvrA binding sites was measured by ChIP-qPCR. Data were normalized against input DNA. Data represent the mean ± standard deviation of the results from three samples. **, *p* < 0.01; ***, *p* < 0.001 by Student’s *t*-test.

PvrA has been characterized as a dual-function transcriptional regulator capable of both activating and repressing target gene expression [30,31]. Sequence alignment of identified multiple conserved PvrA binding motifs (5′-CGGTCA-3′ and its reverse complement 5′-TGACCG-3′) upstream of the *eatR* and *gbdR* coding sequences (Figure 4a).

Promoter activity, EMSA and ChIP-qPCR confirmed direct binding of PvrA to the *eatR* and *gbdR* promoter regions, which suppresses their transcription (Figure 4b–d). As EatR and GbdR are the master activators governing ethanolamine and choline utilization pathways respectively, PvrA inhibits these two nutrient utilization programs via direct transcriptional downregulation of their upstream transactivators.

### 3.5. Fatty Acid-Derived Acyl-CoA Strengthens PvrA-Mediated Repression of Ethanolamine and Choline Utilization

**Figure 5:**
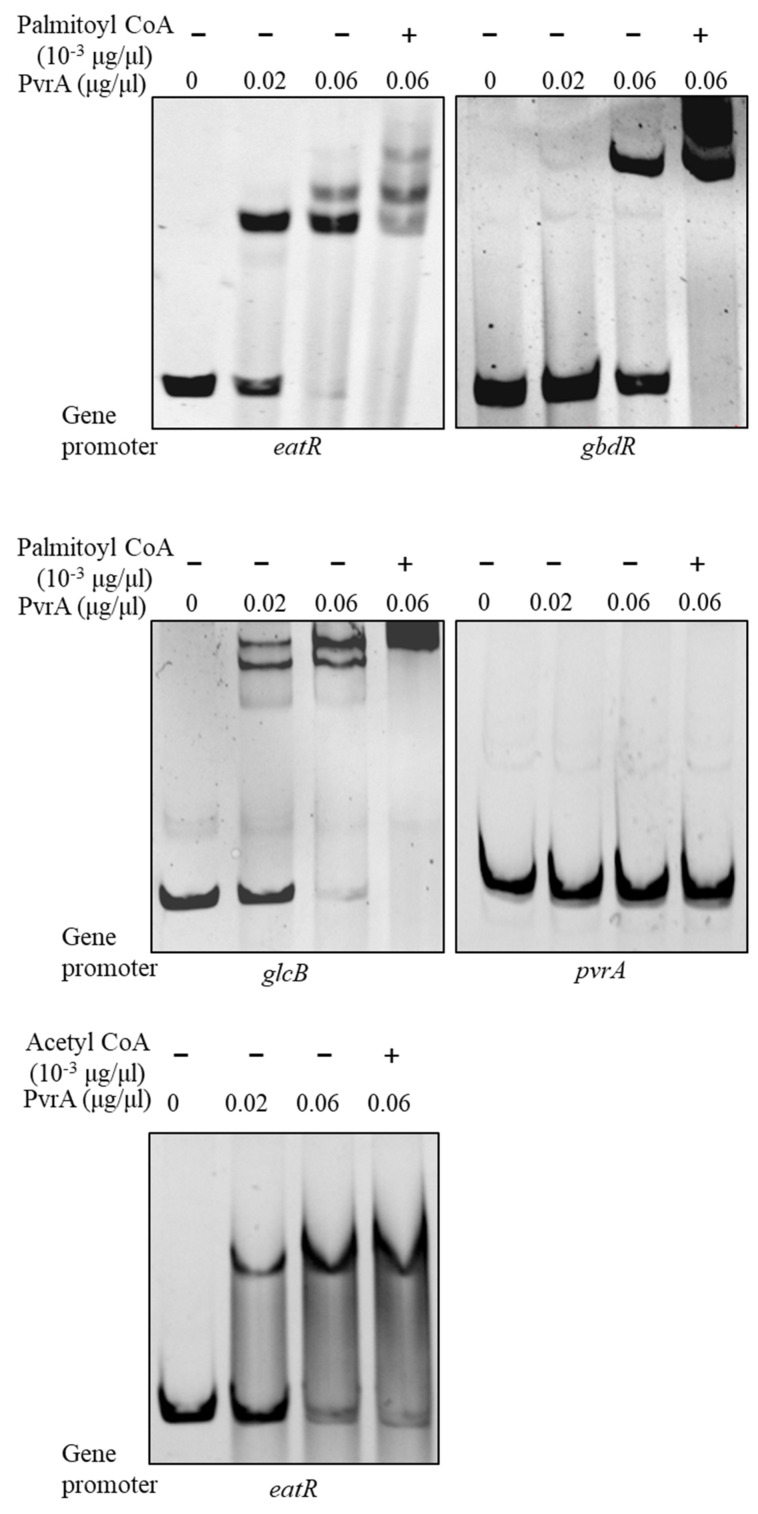
EMSA demonstrating enhanced PvrA-DNA binding in the presence of palmitoyl-CoA. The promoter region of *glcB* and *pvrA* was used as a positive and negative control for PvrA binding respectively. Acetyl-CoA was included to confirm the specific stimulatory effect of palmitoyl-CoA.

The DNA-binding affinity of PvrA is modulated upon interaction with long-chain acyl-CoAs, which act as intracellular signals of fatty acid availability [16,31]. We performed EMSA assays using palmitoyl-CoA and acetyl-CoA to test whether fatty acid-derived signals modulate PvrA-mediated repression of choline/ethanolamine pathways. Addition of palmitoyl-CoA enhanced PvrA binding to the *eatR* and *gbdR* promoters (Figure 5), demonstrating that long-chain fatty acid metabolites amplify PvrA-mediated transcriptional repression of these utilization genes. Collectively, these data indicate that *P. aeruginosa* senses host fatty acids through the PvrA, which simultaneously upregulates fatty acid degradation and utilization genes (*fadD*, *fadE* and *fadB*) and reinforces transcriptional repression of *eatR* and *gbdR*.

## 4. Discussion

Choline primarily contributes to osmoprotection and membrane remodeling. Following uptake, choline is rapidly converted to glycine betaine, a compatible solute that maintains cellular turgor under hyperosmotic stress [24]. Glycine betaine can undergo further demethylation to generate glycine, which feeds into serine biosynthetic pathways [32]. Ethanolamine functions as both carbon and nitrogen sources; its utilization generally requires vitamin B12 as a cofactor [33]. Ethanolamine is cleaved by ethanolamine ammonia-lyase into acetaldehyde and ammonia. Acetaldehyde enters central carbon metabolism via conversion to acetyl-CoA for energy production, whereas ammonia supplies readily assimilated nitrogen for amino acid and nucleotide biosynthesis [34,35].

Prior studies have reported that choline induces transcription of *gbdR* [36,37]. However, our findings show that *gbdR* is repressed when bacteria are cultured with PC, which releases abundant free fatty acids upon hydrolysis. This reveals a regulatory mechanism in which fatty acids override the stimulatory effect of choline. The contribution of ethanolamine catabolism to host colonization varies widely across bacterial species.

Kaval et al. demonstrated that loss of ethanolamine utilization increases gastrointestinal tract colonization in *Enterococcus faecalis*, indicating ethanolamine catabolism may be dispensable or even detrimental to certain pathogenic lifestyles [38]. This counterintuitive outcome was supported by competitive index assays showing that Eut mutants outcompeted wild-type strains in vivo, a finding that contrasts with previous observations in pathogens such as *Salmonella enterica* and enterohemorrhagic *Escherichia coli* (EHEC), where ethanolamine utilization enhances gut colonization and virulence [39,40]. The disparity may be explained by differences in host niche, regulatory architecture, and metabolic context. As reviewed by Kaval and Garsin, ethanolamine utilization is governed by diverse regulatory mechanisms across bacterial lineages [20]. In *Salmonella* and EHEC, the EutR regulator directly links ethanolamine sensing to virulence gene expression, including activation of type III secretion systems and fimbrial genes, thereby integrating metabolism with pathogenicity. In contrast, *E. faecalis* employs the EutV/W two-component system and a riboswitch-based mechanism to control Eut gene expression, with no direct regulatory connection to virulence factors [38]. This difference may explain why ethanolamine catabolism imposes a fitness cost rather than a benefit within the intestinal niche of *E. faecalis*.

In this study, we demonstrate that during acute lung infection, *P. aeruginosa* represses choline and ethanolamine utilization via PvrA-mediated downregulation of *eatR* and *gbdR*, and this repressive effect is enhanced by palmitoyl-CoA derived from fatty acid. Our findings establish a functional regulatory link between fatty acid sensing and suppression of alternative carbon source utilization pathways. The increased colonization observed in *eatR/gbdR* double mutants aligns with the hypothesis that silencing these pathways confers a host adaptation advantage. The mild phenotype and lack of single-mutant effects suggest functional redundancy or niche-specific roles for these pathways. Unlike *Salmonella* and *E. faecalis*, *P. aeruginosa* lacks a complete ethanolamine metabolosome, which may render ethanolamine catabolism inefficient and lead to the accumulation of toxic intermediates such as acetaldehyde [20]. Our prior work fully characterized the in vivo virulence functions of PvrA: loss of *pvrA* drastically attenuates pulmonary colonization during acute infection. PvrA coordinates multiple virulence-associated processes including fatty acid uptake and catabolism, biosynthesis of the PQS signal, and rhamnolipid production. PvrA-mediated repression of *eatR* and *gbdR* represents an additional downstream regulatory axis that contributes to PvrA-dependent host adaptation.

In summary, this study identifies PvrA as a core metabolic regulator that links environmental fatty acid availability to transcriptional suppression of choline and ethanolamine utilization. This regulatory mechanism strengthens bacterial host adaptation and deepens our comprehension of metabolic remodeling during infection. Clinically, our results illuminate a host-adaptive metabolic strategy that may exacerbate chronic *P. aeruginosa* lung infections in CF and COPD patients. Surfactant-rich airways contain abundant phosphatidylcholine and phosphatidylethanolamine, whose breakdown products fuel bacterial proliferation. While PvrA-centered pathways represent potential anti-virulence targets, all therapeutic conjectures remain speculative and demand further experimental validation. Modulating PvrA activity might attenuate bacterial host adaptability, work synergistically with current antibiotics and improve infection clearance, yet the clinical efficacy of such interventions needs systematic preclinical testing in future studies.

## Figures and Tables

**Table 1:** Downregulated genes of ethanolamine or choline uptake and metabolism during infection.

Id	Gene Name	Fold Change	*p* Value	Annotation
PA0031	*betC*	0.302736193	0.001082114	choline sulfatase
PA3236	*betX*	0.13173674	8.73 × 10^−12^	glycine betaine-binding protein
PA4021	*eatR*	0.202646291	3.84 × 10^−28^	transcriptional regulator
PA4022	*hdhA*	0.046519277	1.96 × 10^−36^	aldehyde dehydrogenase
PA4023	*eat*	0.105738726	4.55 × 10^−15^	transporter
PA4024	*eutB*	0.177911391	1.29 × 10^−12^	ethanolamine ammonia-lyase large subunit
PA4025	*eutC*	0.040201168	4.16 × 10^−14^	ethanolamine ammonia-lyase small subunit
PA5372	*betA*	0.446080603	4.62 × 10^−25^	choline dehydrogenase
PA5373	*betB*	0.247112252	5.89 × 10^−99^	betaine aldehyde dehydrogenase
PA5374	*betI*	0.39730866	2.48 × 10^−24^	BetI family transcriptional regulator
PA5378	*cbcX*	0.490372128	8.42 × 10^−10^	hypothetical protein
PA5380	*gbdR*	0.265284293	8.16 × 10^−11^	transcriptional regulator GbdR

**Table 2:** Fatty acid catabolic genes transcriptionally upregulated during acute pulmonary infection.

Id	Gene Name	Fold Change	*p* Value	Annotation
PA3299	*fadD*	3.669716199	1.20 × 10^−109^	long-chain-fatty-acid-CoA ligase
PA3013	*fadA*	8.863443001	3.50 × 10^−135^	3-ketoacyl-CoA thiolase
PA3014	*fadB*	8.785106051	4.14 × 10^−262^	multifunctional fatty acid oxidation complex subunit alpha
PA0508	*fadE*	4.365146956	6.22 × 10^−39^	acyl-CoA dehydrogenase

## Data Availability

The raw data supporting the conclusions of this article will be made available by the authors on request.

## Supplementary Materials

The following supporting information can be downloaded at: https://www.mdpi.com/article/10.3390/pathogens15070680/s1, Table S1: Bacterial strains, plasmids and primers used in this study. Refs. [16,41,42,43] are cited in the Supplementary Material.
