# Supplementary material for: PvrA-Mediated Inhibition of Choline and Ethanolamine Uptake Promotes Pseudomonas aeruginosa Colonization in the Host Environment
Source: Pathogens. 2026 Jun 26;15(7):680. doi: 10.3390/pathogens15070680 (PMC13415123; doi:10.3390/pathogens15070680)
Supplement: Supplementary file 1 [file pathogens-15-00680-s001.zip › pathogens-4258326-supplementary.pdf]

Table S1. Bacterial strains, plasmids and primers used in this study.

Strain/ Plasmid /Primer	Description	Source (Reference)
P. aeruginosa		
PA14	Wild type strain of *Pseudomonas aeruginosa*	[41]
△*pvrA*	PA14 deleted of *pvrA*	[16]
△*eatR*	PA14 deleted of *eatR*	This study
△*gbdR*	PA14 deleted of *gbdR*	This study
△*eatR*/△*gbdR*	PA14 deleted of *eatR* and *gbdR*	This study
E. coli		
DH5*α*	F^- ,φ80d*lacZ*ΔM15,Δ(*lacZYA-argF*)U169, *deoR,recA1,endA1,hsdR17*(rk- ,mk+),*phoA*, *supE44*, *λ-* , *thi-1*, *gyrA96*, *relA1*	TransGen
S17-1	*ecA*, *pro*, *hsdR*, RP4-2-Tc::Mu-Km::Tn7	Stratagene
BL21 (DE3)	F^- *ompT hsdSB* (rB-, mB-) *gal dcm* (DE3)	invitrogen
**Plasmid**		
pUC18T-mini-Tn7T-Gm	mini-Tn7 base vector from insertion into chromosome attTn7 site; Gm^r	[42]
pGEX-6P-1	Expression vector, Amp^r	Novagen
pET28a	Expression vector, Kan^r	Novagen
pDN19lacΩ	Promoterless *lacZ* fusion vector; Tc^r	[43]
P_{eatR}-*lacZ*	pDN19lacΩ with *eatR* promoter region; Tc^r	This study
P_{gbdR}-*lacZ*	pDN19lacΩ with *gbdR* promoter region; Tc^r	This study
pET28a-*pvrA*	Expression *pvrA* with his tag, Kan^r	[16]
**Primer**	**Sequence (5′→3′)**	**Function**
Eco-eatR-UF	CGGAATTCCAGCACCTCTTCCATGAACGCC	eatR deletion
Bam-eatR-UR	CGGGATCCTGTTATGGTTCTCCGCGAGGGAC	eatR deletion
Bam-eatR-DF	CGGGATCCCGGGCGGGACGGCTCCA	eatR deletion
Hind-eatR-DR	CCAAGCTTGGGTCACGCCCCGGACC	eatR deletion
pET28aPvrAF	CCCATGGAGCAGAAAGAGCCTCGCAAAG	*pvrA* expression
pET28aPvrAR	CCTCGAGGGCGCTGGGAGCGTCG	*pvrA* expression
EMeatRF	GGTGGAACGGGGGAACTCGGCGATC	EMSA
EMeatRR	TGTTATGGTTCTCCGCGAGGGAC	EMSA
EMgbdRF	CGACACCGGTGCCGACATG	EMSA
EMgbdRR	TAGGGTGTATCTCCTCACG	EMSA
EMglcBF	CTAGACCAGCTCTGGACCC	EMSA
EMglcBR	TGGCCAACGTGACCGT	EMSA
EMpvrAF	GAGCAGGGCGCTCTCG	EMSA
EMpvrAR	GACTGTCAGGTCAAAATG	EMSA
RTeatRF	GGCTGCTATTTCCTCCGTCGCTTC	RT-PCR
RTeatRR	GACCGAGCAGGTCGTCGTCGAGAC	RT-PCR

RThdhAF	CTATTTCGAAGACATCATGCAG	RT-PCR
RThdhAR	GCTTGATCGCCCTGACCTT	RT-PCR
RTeatF	TGTACCTGTGCATGTGCTTCTCGC	RT-PCR
RTeatR	CGAACAGCGACTCGCAGTAGG	RT-PCR
RTgbdRF	TGGGAATGCCTGGCGGCGATGCAG	RT-PCR
RTgbdRR	GATGCGCTCGTAGATGAACATCTC	RT-PCR
RTcbcXF	ACAAGATCTACGGCATCGAGC	RT-PCR
RTcbcXR	TGGAAGCGGGTATTCATGG	RT-PCR
RTbetAF	AACGGCTACCAGCAGGAAG	RT-PCR
RTbetAR	GTGACCGGGTTGTCGCC	RT-PCR
Eco-P4021-F	CGGAATTCGGTGGAACGGGGGAACTCGGCGATC	Promoter activity
Bam-P4021-R	CGGGATCCTGTTATGGTTCTCCGCGAGGGAC	Promoter activity
Eco-gbdR-F	CGGAATTCGTCGAGTCGTCGCAGTGCTG	Promoter activity
Bam-gbdR-R	CGGGATCCGCGCACCTCGGTGGGTATGA	Promoter activity
